# Chikungunya virus infection in Aruba: Diagnosis, clinical features and predictors of post-chikungunya chronic polyarthralgia

**DOI:** 10.1371/journal.pone.0196630

**Published:** 2018-04-30

**Authors:** Ralph Huits, Jaclyn De Kort, Riemsdijk Van Den Berg, Luis Chong, Achilleas Tsoumanis, Kaat Eggermont, Koen Bartholomeeusen, Kevin K. Ariën, Jan Jacobs, Marjan Van Esbroeck, Emmanuel Bottieau, Lieselotte Cnops

**Affiliations:** 1 Department of Clinical Sciences, Institute of Tropical Medicine, Antwerp, Belgium; 2 Department of Internal Medicine, Horacio Oduber Hospital, Oranjestad, Aruba; 3 Landslaboratorium Aruba, Oranjestad, Aruba; 4 Department of Biomedical Sciences, Institute of Tropical Medicine, Antwerp, Belgium; 5 Department of Biomedical Sciences, University of Antwerp, Antwerp, Belgium; 6 Department of Microbiology and Immunology, University of Leuven, Leuven, Belgium; Emory University School of Medicine, UNITED STATES

## Abstract

**Background:**

Chikungunya virus (CHIKV) emerged in Aruba for the first time in 2014. We studied the clinical presentation of acute CHIKV infection and the contribution of serologic and molecular assays to its diagnosis. In a cohort of confirmed CHIKV cases, we analysed the frequency, duration and predictors of post-chikungunya chronic polyarthralgia (pCHIK-CPA), defined as joint pains lasting longer than 6 weeks or longer than 1 year.

**Methodology:**

Patient sera obtained within 10 days of symptom onset were tested for CHIKV, using an indirect immunofluorescence test for the detection of CHIKV-specific Immunoglobulin M (IgM) and post-hoc, by reverse-transcription polymerase chain reaction (RT-PCR). CHIKV was isolated from selected samples and genotyped. For confirmed CHIKV cases, clinical data from chart review were complemented by a Telephone survey, conducted 18–24 months after diagnosis. When joint pain was reported, the duration, presence of inflammatory signs, type and number of joints affected, were recorded. Joint involvement was scored according to the 2010 ‘American College of Rheumatology/ European League Against Rheumatism’ criteria for seronegative rheumatoid arthritis (ACR-score). Risk factors for pCHIK-CPA were identified by logistic regression.

**Principal findings:**

Acute CHIKV infection was diagnosed in 269 of 498 sera, by detection of IgM (n = 105), by RT-PCR (n = 59), or by both methods (n = 105). Asian genotype was confirmed in 7 samples. Clinical data were complete for 171 of 248 (69.0%) patients, aged 15 years or older (median 49.4 [35.0–59.6]). The female-to-male ratio was 2.2. The main acute symptoms were arthralgia (94%), fever (85%), myalgia (85%), headache (73%) and rash (63%). In patients with arthralgia (n = 160), pCHIK-CPA longer than 6 weeks was reported by 44% and longer than 1 year by 26% of cases. Inflammatory signs, stiffness, edema and redness were frequent (71%, 39% and 21%, respectively). Joints involved were knees (66%), ankles (50%), fingers (52%), feet (46%), shoulders (36%), elbows (34%), wrists (35%), hips (31%), toes (28.1%) and spine (28.1%). Independent predictors of pCHIK-CPA longer than 1 year were female gender (OR 5.9, 95%-CI [2.1–19.6]); high ACR-score (7.4, [2.7–23.3]), and detection of CHIKV-RNA in serum beyond 7 days of symptom onset (6.4, [1.4–34.1].

**Conclusions:**

We identified 269 CHIKV patients after the first outbreak of Asian genotype CHIKV in Aruba in 2014–2015. RT-PCR yielded 59 (28%) additional CHIKV diagnoses compared to IgM antibody detection alone. Arthralgia, fever and skin rash were the dominant acute phase symptoms. pCHIK-CPA longer than 1 year affected 26% of cases and was predicted by female gender, high ACR-score and CHIKV-RNA detection beyond 7 days of symptom onset.

## Introduction

The word ‘chikungunya’ was used by the Makonde people of Southern Tanzania to describe the severe joint pains that literally bent the affected patients’ posture. The causative agent, chikungunya virus (CHIKV), was first isolated during an explosive outbreak in East Africa in 1952 [1] [2]. It is a positive-sense single stranded RNA virus that belongs to the genus *alphavirus* of the family *Togaviridae*. The virus is transmitted by mosquitoes, and *Aedes aegypti* was identified as the primary vector [3]. Three genotypes of CHIKV have been identified: West African, Eastern/Central/Southern African (ECSA) and Asian [4].

After the isolation and description of CHIKV, spill-over infection from sylvatic transmission cycles and small scale epidemics were reported from African and South-East Asian countries in the second half of the twentieth century [5]. However, since 2005 ECSA and Asian genotype CHIKV has spread across continents in outbreaks that involved millions of people and put millions more at risk globally [6][7]. The emergence of CHIKV as a global pathogen has been attributed to multiple factors. Urbanization in countries where CHIKV was endemic, allowed convergence of human and vector populations. Increased air travel permitted frequent exposure and rapid spread of susceptible human populations to the virus. International trade, climate change and lack of adequate vector control measures contributed to suitable environments for geographic expansion of its vector species [8]. Finally, showcasing the nature of evolution, CHIKV adapted to replication in *Aedes albopictus* by a single mutation in the envelope protein gene (E1-A226V) in the ECSA genotype. The resulting increase in infectivity of this highly competent vector led to enhanced transmission during the 2005–2006 outbreak on Reunion island [9].

Signs of acute CHIKV infection other than arthralgia, are fever, myalgia and rash. Clinical distinction from other arthropod-borne viral illness is not possible. Still, diagnosing CHIKV infection at an early stage is important, as the acute febrile illness is frequently followed by post-chikungunya chronic polyarthralgia (pCHIK-CPA). The chronic, symmetric joint pains of pCHIK-CPA may resemble seronegative rheumatoid arthritis [10,11].

Aruba is an island in the Lesser Antilles of 180 square kilometres, north of Venezuela. Its population of approximately 100,000 inhabitants is of mixed descent, originating from Europe, Latin America, and Africa [12].

Our study of the first CHIKV outbreak in Aruba had three objectives. First, we evaluated the contribution of serologic and molecular testing to the diagnosis of CHIKV in the acute phase. Second, we performed a retrospective assessment of the clinical presentation of CHIKV infection in adults. Third, we identified risk factors associated with pCHIK-CPA.

## Methods

**Fig 1:**
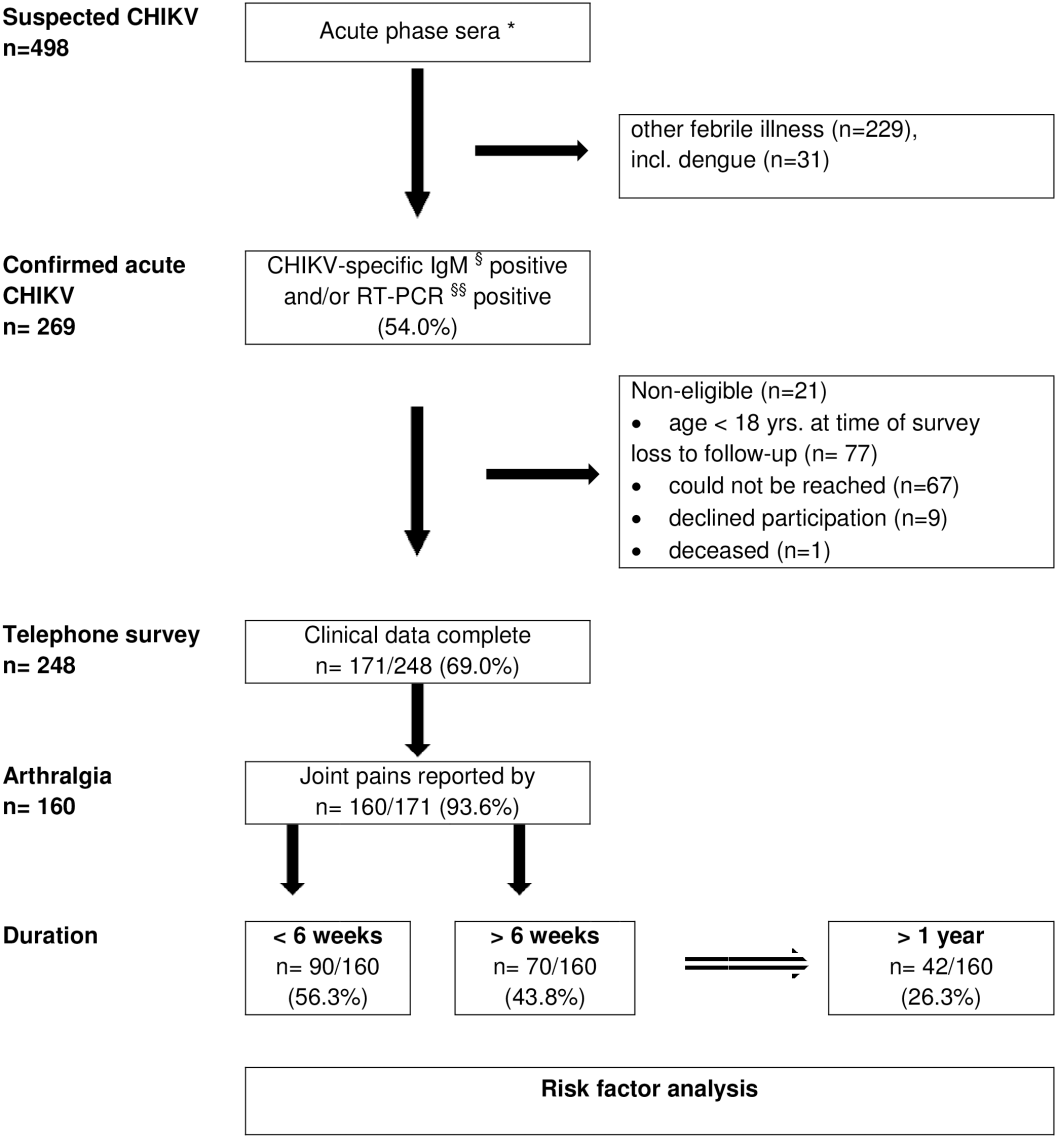
Study design. * Acute phase sera refers to all available sera collected within 10 days of symptom onset. ^§^ Positive IgM: Immunoglobulin M (IgM) detected in indirect immunofluorescence assay (IFA) (Anti-CHIKV IIFT IgM/IgG, Euroimmun, Lübeck, Germany) according to manufacturer’s instructions. ^§§^ positive RT-PCR: CHIKV RNA detected in a RT-PCR targeting the NSP-1 gene using primers and probes that detect CHIKV strain (LR2006_OPY_1, Indian Ocean Islands and the African prototype (S27, general strain)).

During the 2014–2015 CHIKV outbreak, Aruban patients were evaluated for CHIKV at their physician’s discretion, using a serum sample obtained within 10 days post symptom onset (DPSO) (Fig 1). The laboratory diagnosis of acute CHIKV infection in a single sample requires a testing algorithm that combines an assay for CHIKV-specific Immunoglobulin M (IgM) antibody detection and a molecular method for CHIKV-RNA detection [13,14].

### Diagnostics

Acute CHIKV infection in our study was defined by either or both positive results of CHIKV-specific IgM in an IgM/IgG indirect immunofluorescence assay (IFA) (Anti-CHIKV IIFT IgM/IgG, Euroimmun, Lübeck, Germany) according to manufacturer’s instructions, and CHIKV-specific real-time reverse-transcription polymerase chain reaction (RT-PCR). Sera with positive IgG, but negative IgM result in IFA and negative RT-PCR were not considered acute infections. Convalescent sera to assess seroconversion were not available. The Landslaboratorium was the only diagnostic facility on the island that tested for CHIKV, using IFA. All sera were stored at the Landslaboratorium at -80°C, until shipment to the Institute of Tropical Medicine (ITM, Antwerp Belgium), where CHIKV-specific RT-PCR was performed as described previously. Briefly, a specific part of the nonstructural protein 1 (NSP-1) gene was amplified on the LightCycler 96 (Roche) according to the protocol described by Van den Bossche et al., with primers and probes detecting the African and Asian CHIKV strains[15,16]. Real-time RT-PCR was run for 50 cycles, and any Ct-value < 50 was considered positive. Isolation of CHIKV by inoculation onto Vero cells (ATCC^®^ CCL-81^™^) was attempted from samples with Ct-values below 30. The E1-region of the isolates was genotyped (Sanger sequencing method) [17].

Because dengue virus (DENV) is endemic in Aruba, a DENV multiplex RT-PCR to differentiate the four serotypes (DENV1-4) was performed with an in-house test (adapted from ref. [18]) on the sera of confirmed acute CHIKV cases, to exclude co-infection.

### Data collection

Clinical data from the laboratory request forms (gender, age and date of symptom onset- defined as 0 DPSO) were complemented by a structured telephonic questionnaire. The interviews were conducted by a trained infectious disease physician, 18–24 months after diagnosis, after obtaining oral consent. Patients with confirmed CHIKV aged 18 years or older, were eligible for interviewing. Participants were excluded after three failed attempts to contact them. Additional data obtained included comorbidities (diabetes, obesity, history of cardiovascular, respiratory or pre-existing rheumatological conditions, osteoporosis, osteoarthritis) and symptoms in the acute phase (fever, rash, arthralgia, myalgia, headache, respiratory or gastro-intestinal symptoms, difficulty sleeping or concentrating). When arthralgia was reported, the pattern of joint involvement was systematically scored in accordance with the 2010 American College of Rheumatology/ European League Against Rheumatism (ACR/EULAR) criteria for seronegative rheumatoid arthritis [19]. Briefly, the pattern of distribution of affected joints was scored based on the number and type of joints involved. ‘Large joints’ refers to shoulders, elbows, hips, knees, ankles and spine; ‘Small joints’ refers to the metacarpophalangeal, interphalangeal, metatarsophalangeal and wrist joints. We assigned low ACR-scores ‘1, 2 and 3’ to ‘one large joint, 2–10 large joints, and 1–3 small joints (with or without involvement of large joints), respectively. High ACR scores ‘4 and 5’ were assigned when involvement of ‘4–10 small joints (with or without involvement of large joints) or involvement of more than 10 joints (involving at least one small joint)’ were recorded. Symmetry, defined as bilateral involvement of at least one joint region, was assumed in all cases, unless participants explicitly reported involvement of a single joint [11]. Reported edema, redness and morning stiffness that lasted more than 30 minutes were recorded as clinical signs of inflammatory arthropathy. Self-reported duration of pain in adults with confirmed CHIKV was scored in six categories: shorter than 2 weeks, 2–6 weeks, 3–6 months, 6 weeks-3 months, 6–12 months or longer than 12 months. pCHIK-CPA was defined as joint pain lasting more than 6 weeks.

The frequency of medical consultations and duration of absenteeism (less than one week, 1–2 weeks, 2–4 weeks, more than 4 weeks or still not able to work) were recorded.

### Outcomes

As primary outcomes, we recorded the relative contribution of RT-PCR and IgM antibody assays to the compound diagnosis of acute CHIKV infection per day of sampling post symptom onset, the duration of pain in adults with confirmed CHIKV (shorter than 6 weeks, longer than 6 weeks and longer than one year) and potential risk factors for pCHIK-CPA, lasting more than 6 weeks or more than 1 year. Secondary outcomes were the frequency of follow-up medical consultations and the duration of absenteeism.

### Statistics

Continuous variables were summarized as medians and inter-quartile ranges. Categorical variables were expressed as counts and percentages. Chi-square test or Fisher’s exact test were used to test for association between categorical variables. Odds ratios (OR) with 95%-CI were calculated to identify risk factors in univariate logistic regression models. When significant at 10% level, a multivariate regression model was fitted to adjust for confounding and multiple predictors. The final model was selected using stepwise backward elimination with likelihood ratio test as the comparison test. All analyses were done in R (version 3.4.1).

### Ethics statement

Ethics approval for this study was obtained from the Institutional Review Board at the Institute of Tropical Medicine and Ethics Committee of the University Hospital in Antwerp, Belgium. Approval for the collection of clinical data by telephonic survey after oral consent was obtained from the Board of the Horacio Oduber Hospital in Aruba. All subjects were 18 years or older when interviewed by a physician, who proceeded only after oral consent and documenting the obtained consent on the questionnaire form (See Supporting Information).

## Results

**Fig 2:**
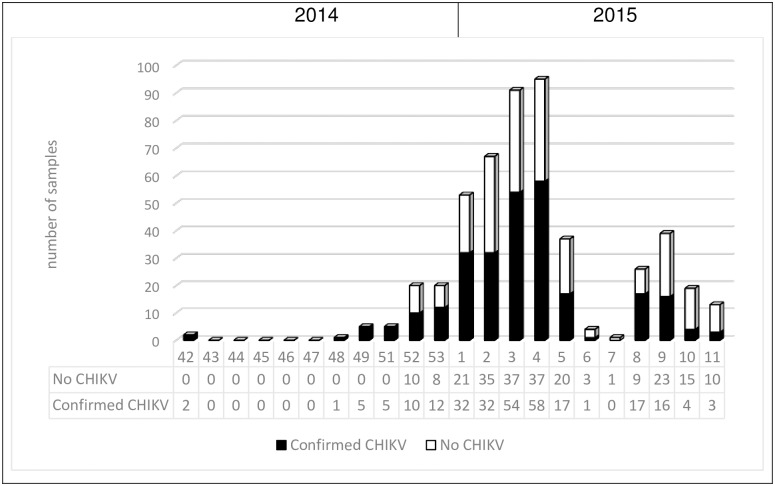
Number of samples collected for chikungunya diagnostics per epidemiological week, during the outbreak in Aruba from October 2014 –March 2015 (n = 498). Note: epiweek 6 and 7 coincided with Carnival, Aruba’s public holiday.

Sera of 498 patients with clinical suspicion of acute CHIKV infection were collected within the first 10 DPSO, from October 2014 (epiweek 42) to April 2015 (epiweek 11) (Fig 2). In total, acute CHIKV infection was diagnosed in 269 (54.0%) patients, 175 female and 94 male (female-to-male ratio 1.9).

CHIKV-specific IgM was positive in 210 patient sera, in 105 of which RT-PCR was also positive. In addition, RT-PCR was positive in 59 sera for which CHIKV-specific IgM was negative. CHIK-specific IgG was detected in 122 of the 210 patients with positive IgM result, and in 56 of the 164 RT-PCR positive samples. CHIKV-specific IgG alone was detected in 9 out of 498 sera (1.8%), all collected from January 2015 onwards.

**Fig 3:**
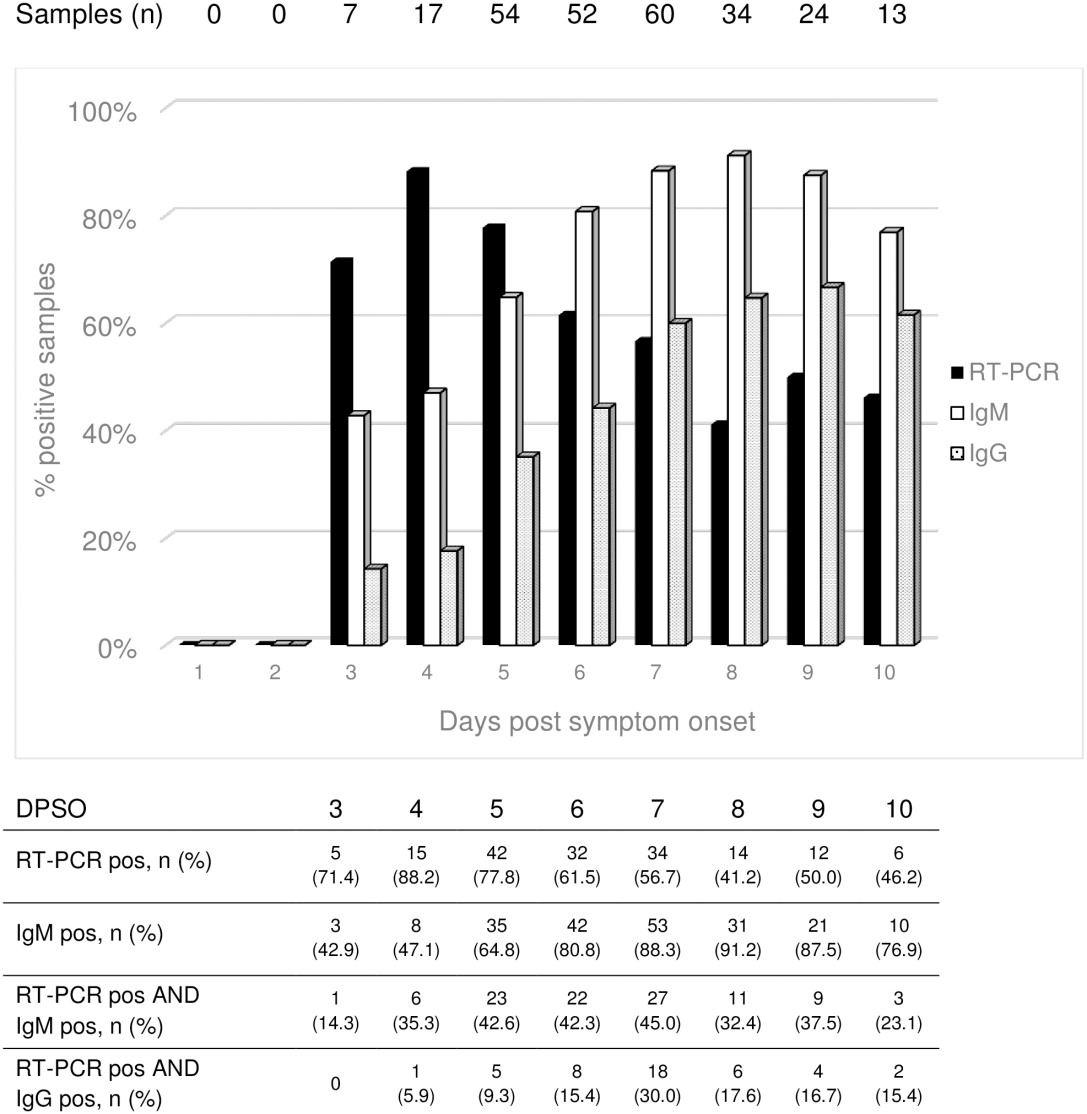
Proportions of positive chikungunya virus-specific RT-PCR, IgM and IgG antibodies (IFA) per day of sampling post symptom onset *. * Note: exact date of sampling was available for 261 patients with confirmed CHIKV infection.

The exact date of sampling post symptom onset was available for 261 out of 269 sera (97.0%) with confirmed CHIKV infection and the respective proportions of positive IFA and RT-PCR results was calculated on this number (Fig 3). RT-PCR was still positive in up to 50% of the sera collected between DSPO 8–10.

DENV-RNA was not detected in any of the 269 sera with confirmed acute CHIKV. In 31 of 229 sera without CHIKV (13.5%), evidence of acute DENV infection was found. DENV-specific RT-PCR was positive in 6, and DENV-specific IgM was positive in 25.

Isolation of CHIKV by inoculation onto Vero cells was attempted from 13 samples with Ct-values ranging from 17.3 to 31.0, and was successful in 8 (Ct-values 17.3–28.7). The E1-region (position 9994–11310) of 7 isolates was genotyped by Sanger sequencing; all 7 clustered with the Asian-Caribbean strain.

Follow-up interviews were conducted from June 2016 to June 2017. At that time, 21 of 269 confirmed CHIKV cases were younger than 18 years, leaving 248 eligible for clinical reassessment by telephonic survey. Hundred seventy-one of 248 patients (69.0%) were interviewed. Sixty-seven were not contacted after 3 attempted calls, 9 declined to be interviewed, and one had died of causes unrelated to CHIKV infection. Their median age was 50.2 years (IQR 35.9–60.2 years). Hundred seventeen respondents were female, 54 were male (female-to-male ratio 2.2). The non-respondents had comparable distribution of age (p-value = 0.12, Chi-square test) and gender (p-value = 0.34, Mann-Whitney U test). Self-reported comorbidities among 171 respondents are reported in Table 1.

**Table 1:** Characteristics of 171 patients with confirmed chikungunya infection.

Age (median yrs., [IQR])	49.4	[35.0–59.6]
< 18 yrs.	11	(6.4)
18–40 yrs.	40	(23.4)
41–60 yrs.	78	(45.6)
> 60 yrs.	42	(24.6)
Gender	n	(%)
Female	117	(68.4)
Male	54	(31.6)
Female-to-male ratio	2.2	
Medical history	n	(%)
Diabetes	14	(8.2)
Obesity	25	(14.6)
Cardiovascular	20	(11.7)
Respiratory	11	(6.4)
Rheumatological	4	(2.3)
Osteoporosis	5	(2.9)
Osteoarthritis	11	(6.4)
Other	38	(22.2)
Symptoms	n	(%)
Arthralgia	160	(93.6)
Fever	146	(85.4)
Myalgia	145	(84.8)
Headache	124	(72.5)
Skin rash	107	(62.6)
Respiratory symptoms ^§^	35	(20.5)
Gastro-intestinal symptoms ^§§^	52	(30.4)
Dizziness	48	(28.1)
Bleeding	4	(2.3)
Sleeping disorder	59	(34.5)
Concentration disorder	39	(22.8)
Absenteeism	N	(%)
< 1 week	41	(24.0)
1–2 weeks	33	(19.3)
2–4 weeks	18	(10.5)
> 4 weeks	8	(4.7)
Still not able to work	1	(0.6)
Health visit (physician)	82	(48.0)

^§^ Nausea, diarrhoea, vomiting;

^§§^ cough, sore throat, dyspnoea.

Values in n (percent) unless otherwise noted.

Symptoms during the acute phase confirmed a pattern of CHIKV infection as an acute febrile illness with arthralgia and myalgia as the most prominent clinical features (Table 1). None of the CHIKV-infected patients were hospitalized and no CHIKV-related mortality was recorded.

Arthralgia was reported by 160 (93.6%) patients. Among these 160 patients, joint involvement was symmetrical in 156 (97.5%). The affected joints are listed in Table 2. Seventy-eight patients (48.8%) were assigned a low ACR-score and 82 patients (51.3%) had a high ACR-score. Involved joints were stiff, edematous and red in 71.3%, 38.8% and 21.3%, respectively (Table 2).

**Table 2:** Pattern of joint involvement, inflammatory symptoms and pain duration in chikungunya virus infected patients presenting with arthralgia (n = 160).

Affected joints		n	(%)
shoulder		57	(35.6)
elbow		54	(33.8)
wrist		56	(35.0)
finger		83	(51.9)
hip		49	(30.6)
knee		105	(65.6)
ankle		80	(50.0)
feet		73	(45.6)
toes		45	(28.1)
spine		45	(28.1)
ACR-score ^#^		N	(%)
low	1. (1 large joint)	4	(2.5)
	2. (2–10 large joints)	42	(26.3)
	3. (1–3 small joints)	32	(20.0)
high	4. (4–10 small joints)	43	(26.9)
	5. (> 10 joints)	39	(24.4)
Inflammatory symptoms		n	(%)
redness		34	(21.3)
edema		62	(38.8)
stiffness		114	(71.3)
Duration of joint pains		n	(%)
< 2 weeks		59	(36.9)
2–6 weeks		31	(19.4)
6 weeks–3 months		11	(6.9)
3–6 months		8	(5.0)
6–12 months		9	(5.6)
> 12 months		42	(26.3)

^#^ The ACR-score was assigned according to type and number of joints involved, based on the 2010 ‘American College of Rheumatology/ EUropean League Against Rheumatism’ criteria for seronegative rheumatoid arthritis [19]. ‘Large joints’ refers to shoulders, elbows, hips, knees, ankles and spine. ‘Small joints’ refers to the metacarpophalangeal, interphalangeal, metatarsophalangeal and wrist joints. Low ACR-scores were: ‘one large joint’ (1), ‘2–10 large joints’ (2) and ‘1–3 small joints (with or without involvement of large joints)’ (3). High ACR scores were assigned to involvement of ‘4–10 small joints (with or without involvement of large joints)’ (4), or ‘involvement of more than 10 joints (involving at least one small joint)’ (5).

pCHIK-CPA longer than 6 weeks was recorded in 70/160 (43.8%) and longer than 1 year in 42/160 (26.3%) of cases. Adjusted risk factors from the multivariate analysis for pCHIK-CPA longer than 6 weeks were female gender (OR 3.4, 95%-CI [1.6–8.0]), obesity (4.8, [1.8–14.7]) and concentration disorder (4.7, [2.1–11.1]). Independent risk factors for pCHIK-CPA longer than 1 year were female gender (5.9, [2.1–19.6]), and a high ACR-score (7.4 [2.7–23.3]). For patients tested at 8, 9 or 10 DPSO (n = 42), the OR for pCHIK-CPA longer than 6 weeks was 4.0 ([1.1–17.6]), and 6.4 ([1.4–34.1]) for pCHIK-CPA longer than 1 year when RT-PCR result was positive for CHIKV-RNA, compared to patients with a negative RT-PCR beyond 7 DPSO (Table 3).

**Table 3:** Risk factors for chikungunya virus-associated long-term polyarthralgia (n = 160).

	Pain duration						
	< 6 weeks (n = 90)	> 6 weeks (n = 70)			> 1 year (n = 42)		
	n = 90 (100%)	n = 70 (100%)	OR [95%-CI]		n = 42 (100%)	OR [95%-CI]	
Risk factors			*Univariate*	*Multivariate*		*Univariate*	*Multivariate*
Age, yrs (IQR)	50 (35–63)	50 (37–56)			49 (37–54)		
≤ 40	25 (27.8)	20 (4.3)	-		13 (31.0)	-	
> 40	65 (71.4)	50 (24.3)	1.0 [0.5–2.0]		29 (69.0)	1.1 [0.5–2.5]	
Female gender	52 (57.8)	57 (81.4)	3.2 [1.6–6.9]	3.4 [1.6–8.0]	36 (85.7)	4.4 [1.8–12.5]	5.9 [2.1–19.6]
Obesity	6 (6.6)	17 (24.3)	4.5 [1.8–13.1]	4.8 [1.8–14.7]	10 (23.8)	4.4 [1.5–13.8]	
Diabetes	8 (8.8)	4 (5.7)	0.6 [0.2–2.1]		2 (4.8)	0.5 [0.1–2.2]	
Rheumatol. condition	3 (3.3)	1 (1.4)	0.4 [0.0–3.4]		1 (2.4)	0.7 [0.0–5.7]	
Osteoporosis	5 (5.5)	0			0		
Osteo-arthritis	5 (5.5)	4 (5.7)	1.0 [0.3–4.1]		3 (7.1)	1.3 [0.3–5.7]	
Sleep *	25 (27.4)	32 (45.7)	2.2 [1.1–4.3]		22 (52.4)	2.9 [1.3–6.2]	
Concentration *	12 (13.2)	26 (37.1)	3.8 [1.8–8.6]	4.7 [2.1–11.1]	17 (40.5)	4.4 [1.9–10.7]	
Redness	17 (18.7)	17 (24.3)	1.4 [0.7–3.0]		9 (21.4)	1.2 [0.5–2.9]	
Edema	26 (28.9)	36 (51.4)	2.5 [1.3–4.9]		25 (59.5)	3.6 [1.7–7.9]	
Stiffness	58 (64.4)	56 (80.0)	2.2 [1.1–4.6]		34 (81.0)	2.3 [1.0–5.9]	
Finger	39 (43.3)	44 (62.9)	2.2 [1.2–4.1]		31 (73.8)	3.7 [1.7–8.5]	
High ACR-score	37 (41.1)	45 (64.3)	2.6 [1.4–5.0]	2.9 [1.4–6.1]	33 (78.6)	5.3 [2.3–12.9]	7.4 [2.7–23.3]
RT-PCR positive > 7 DPSO^¶^	4/20 (20.0)	11/22 (50.0)	4.0 [1.1–17.6]		8/13 (61.5)	6.4 [1.4–34.1]	

To control for confounding we performed Stepwise Logistic Regression, using backward elimination for any variable (including age) whose univariate test p-value < 0.10.

* Sleep and concentration disorder were identified as risk factors for arthralgia; however, in our study it was not possible to distinguish whether these were predictors or consequences.

DPSO denotes days post symptom onset.

^¶^ The Odds Ratios were calculated for 42 of 160 patients, whose sera were collected after 7 DPSO.

Patients with pCHIK-CPA longer than 6 weeks or longer than 1 year consulted their physician more frequently (OR 1.8, [1.0–3.5] and 2.2, [1.0–4.8], respectively). Hundred-and-one out of 171 interviewed patients (59.1%) reported absenteeism. Ninety-five out of 101 (94.1%) who took sick leave reported arthralgia. Four patients without arthralgia were absent from work for less than a week, two less than 2 weeks. Absenteeism longer than 2 weeks was recorded in 27.1% of patients with pCHIK-CPA longer than 6 weeks and in 31.0% of those with pCHIK-CPA longer than 1 year. Compared to patients without pCHIK-CPA, the OR for sick leave more than 2 weeks was 3.8 ([1.5–10.1]) when joint pains lasted more than 6 weeks, and 4.6 ([1.6–13.1]) when joint pains lasted more than one year.

## Discussion

The study of confirmed CHIKV cases during the 2014–2015 outbreak in Aruba enabled us to assess the relative contribution of serologic and molecular assays to the diagnosis of acute CHIKV, the clinical presentation and risk factors for pCHIK-CPA. CHIKV infection was confirmed in 269 out of 498 (54%) symptomatic patients, who had been referred by their family physicians for laboratory confirmation of suspected acute infection. This high frequency of confirmed CHIKV infection probably reflected the high prevalence of CHIKV during the outbreak and the rather good selection of candidates for testing based on clinical characteristics. However, we highlight that the absence of CHIKV nucleic acid detection assays in diagnostic facilities on the island led to substantial underdiagnosis in the acute phase of infection. This suggests that the incidence rates of suspected and confirmed CHIKV cases in Aruba as reported to PAHO (443 and 863 per 100.000, respectively), probably underestimated the true magnitude of the outbreak [20,21].

After a first introduction of CHIKV in immunologically naïve populations, reported attack rates vary widely in seroprevalence studies (10 to 75%) [7]. These high attack rates have been attributed to high levels of viremia following CHIKV infection, to short incubation periods (both intrinsic, *i*.*e*. in the human host, and extrinsic, *i*.*e*. in the mosquito vector), and to the presence of competent vector populations [22].

By our study design, we could not identify asymptomatic infections or symptomatic individuals who did not seek consultation. In contrast to Flavivirus infections, asymptomatic CHIKV infections appear less frequent. Ranges from 3–28% have been reported from outbreaks involving ECSA genotype CHIKV [23–26]. However, in the study by Gay *et al*. from Saint Martin 39.0% of CHIKV infections were asymptomatic [27]. Not all symptomatic CHIKV infected patients seek medical attention. In Puerto Rico, laboratory evidence of recent CHIKV infection was found in 28% household contacts of confirmed CHIKV cases who did not consult a physician, even though 84% reported compatible symptoms [26].

In the Caribbean, early estimates of CHIKV incidence ranged from 52 to 115 cases per 1,000 after the emergence of an Asian genotype CHIKV in 2013 [28]. Seroprevalence data obtained seven months after the outbreak in Saint Martin suggested that 16.9% of the population had been infected [27]. Assuming an attack rate of 10% in Aruba’s population of 100,000, we estimate that our cohort represents approximately 2.5% of the total number of CHIKV cases during the 2014–2015 outbreak.

### Diagnostics

Using IFA, CHIKV-specific IgM was detected in the sera of 210 of 269 confirmed acute cases in our cohort. Ninety-seven percent of samples were collected at 3 DPSO or later. Although plaque reduction neutralization tests to increase specificity were not performed, false positive IFA results are unlikely, because alphavirus outbreaks had not been recorded previously on Aruba, and because of the high analytical specificity of the IFA [29]. The accuracy of CHIKV diagnostic tests is obviously correlated to the time kinetics of viremia and specific antibody responses in human CHIKV infection.

### CHIKV-specific IgM

The sensitivity of the IgM in the IFA only approximated 80% or higher after 6 DPSO (Fig 3). Post-hoc CHIKV-specific RT-PCR was positive in the sera of 164 of 269 (61%) cases. In the acute phase, the diagnostic yield additional to IgM detection alone was 28.1%. Other studies reported detection of IgM (and IgG) in 100% of cases from 5 or 6 DPSO onward [16][30].

Variation in epitope recognition between the epidemic strains might explain differences in sensitivity of tests that detect IgM. This was observed in an evaluation of commercial serological assays (including the Euroimmun IFA) between the two subsequent ECSA genotype CHIKV outbreaks in Singapore, one of which involved the E1-A226V mutation [31]. Variation between the Asian genotype CHIKV in Aruba and the ECSA genotype CHIKV that is used as an antigen substrate in the IFA, may have affected IgM detection in our cohort.

Another hypothesis to explain the reduced IgM detection rates in our cohort at 7 DPSO could be slower induction of CHIKV-specific antibody responses following infection with Asian-Caribbean genotype CHIKV compared to epidemic strains in previous reports because of differences in immunogenicity [32].

### CHIKV-specific IgG

We detected CHIKV-specific IgG antibodies from 3 DPSO onwards. Detection of IgG this early is unexpected. Published data suggest that IgG does not appear until 6 DPSO, usually 1–2 days after IgM [13,16,30]. The time-kinetics of the CHIKV-specific IgG response in relation to IgM and RNA results seems compatible with acute infection (Fig 3). Isolated IgG-positive results (*i*.*e*. in sera with negative RT-PCR and IgM results) were a rare finding in our cohort, and occurred only at the height of the epidemic or later. We therefore believe that all positive antibody detection results indicate recent CHIKV infection during the outbreak we describe here.

### CHIKV-specific RT-PCR

The highest proportion of viremic cases (88%) was found at 4 DPSO. CHIKV RNA was detected in spite of the presence of IgM and IgG in up to 45% and 30% of sera, respectively. This is an unexpected finding, for experimental observations had suggested that CHIKV-specific antibodies had strong neutralizing activity in vitro and even therapeutic potential against CHIKV infection in mice [33]. Jain *et al*. postulated that appearance of CHIKV-specific IgG is required to neutralize the virus [34]. In accordance, previous observations of CHIKV infection in humans coupled the development of a CHIKV-specific antibody response to a rapid decrease in viremia [13,30,31]. In studies from CHIKV outbreaks that employed both type of tests throughout the acute phase of infection, combined positive results for both RT-PCR and antibody detection based assays do not appear frequent, i.e. 1–12%. [35–38]. However, our findings are in line with an analysis at California’s National Reference Laboratory, that detected CHIKV-RNA in 34% and 36% of 376 IgM and IgG positive sera, respectively [39]. Sequencing was not performed in that study, but Asian genotype was assumed since samples were obtained from travellers returning from the Caribbean in 2014.

Interestingly, 40 to 50% of patients in our cohort were viremic beyond 7 DPSO. This contrasts with frequent reports of CHIKV-RNA declining to undetectable levels within a week [16,35,40–43]. One small study from Singapore found viremia to persist for more than a week in 30% of cases [44]. In a large cohort from India, viral RNA was detected in patients who were recruited as late as 12 DPSO [34]. In a Thai study, quantitative RT-PCR detected viremia up to 6 DPSO, but when using a second, nested RT-PCR protocol, CHIKV-RNA was found up to 9 DPSO in all of 45 patients [30]. Detection of viral RNA depends on the sensitivity of the assays employed for nucleic acid detection. Variation in CHIKV genotypes or epidemic strains might account for observed differences in viral kinetics between outbreaks.

### Clinical presentation

Arthralgia, fever, myalgia, headache and skin rash were the most frequent acute phase symptoms reported by CHIKV infected patients in our Telephone survey (Table 1). Many patients complained of dizziness, but no other neurological symptoms were recorded. Sleeping and concentration disorders were recorded frequently, but it is impossible to determine whether they were caused by the infection or resulted from illness and pain. The clinical presentation in this study is largely consistent with reports from recent outbreaks, including those caused by strains derived from the Asian CHIKV lineage that affected territories in the Caribbean and South and Central America [43,45–48]. A notable difference is that as many as 20% of patients in our adult study population experienced cough, sore throat or dyspnoea. Respiratory symptoms associated with CHIKV infection have not been reported consistently, but occurred in 23–30% of cases in epidemics in the Philippines, Trinidad and Tobago, and the Dominican Republic [36,38,49]. Critical illness and death due to pneumonia and respiratory failure were reported in ECSA genotype CHIKV-infected patients during the outbreak at Réunion [50]. It remains unclear whether these cases were caused directly by CHIKV, or by co-infection with respiratory pathogens to which CHIKV infection may predispose [51].

In line with published data from the 2014 Asian lineage outbreak in the Caribbean, no CHIKV-associated mortality was recorded in our series. Mortality resulting from CHIKV infection is thought to be rare and restricted to patients with atypical presentations or comorbidities, although some authors reported case-fatality ratios of up to 5% [6,52,53].

### Arthralgia

The dominant clinical symptom in confirmed CHIKV infection was joint pain. Inflammatory characteristics such as morning stiffness, edema and redness were commonly reported. As a rule, multiple joints were symmetrically affected (resulting in a high ACR-score), with the fingers, knees, ankles and feet most commonly involved. pCHIK-CPA occurred frequently.

Previous reports vary greatly in mode of assessment, description, follow-up and classification of CHIKV-associated joint pains [10]. After clinical evaluation, pCHIK-CPA has been diagnosed as arthritis, spondyloarthritis, tenosynovitis, fibromyalgia and arthralgia syndrome among others [54–57]. The pathogenesis of CHIKV-associated arthropathy remains incompletely understood. The pattern of leucocyte infiltration, cytokine production, and complement activation in CHIKV-infected joints is similar to that found in (seronegative) rheumatoid arthritis [22,58]. Clinically, inflammatory joint destruction by synovitis, erosions and joint space narrowing has been well documented [57,59].

The estimates of long-term arthralgia occurrence after CHIKV vary widely [10,60]. Studies in mice suggest that the Asian CHIKV lineage from the Caribbean induces less severe joint pathology than the ECSA genotype from Réunion [61]. Other sources of variation in disease severity between outbreaks may be differences in host response. Variation is certainly affected by differences in study design, such as cohort size, patient selection, mode and timing of assessment of joint pains, and length of follow-up. Previously, arthralgia was found to persist in 57 to 67% of CHIKV infected patients following the ECSA genotype outbreaks in Réunion and Europe [55,62–65]. These percentages, from studies with an approach and length of follow-up comparable to ours, were supported by a prospective study of viremic patients from Réunion [56]. However, well-designed studies from Sri Lanka and India involving ECSA genotype, found rheumatic pains to last considerably shorter (4.1 and 17.8% at 12 months) [66,67]. In a study of 437 patients who did not have prior joint pains from India, rheumatologists diagnosed 57% with postviral polyarthralgia, 22% with postviral inflammatory polyarthritis and 1.4% with rheumatoid arthritis, 15 months following CHIKV infection [68].

Only a few estimates of chronic sequelae of CHIKV infection in the Americas have been published. In Colombia, Rodriguez-Morales et al. found that polyarthralgia persisted during a median follow-up of 21 weeks in 44.3% of 131 infected patients [69]. In Suriname, 22.2% of 180 symptomatic and viremic CHIKV-patients in prospective follow-up, still reported arthralgia at 6 months [48].

Our data on pCHIK-CPA are consistent with these earlier data from Asian genotype CHIKV outbreaks in the Americas. They are also consistent with a meta-analysis that conservatively estimated the proportion of patients with chronic inflammatory rheumatism following CHIKV-infection at 25%, and the percentage developing arthritis at 14% [60].

### Predictors of CHIKV-associated long-term arthralgia

Analysing our clinical data, three independent risk factors for arthralgia lasting longer than 6 weeks held significance in multivariate analysis: female gender, obesity and a high ACR-score. Female gender and a high ACR-score also predicted pCHIK-CPA longer than one year. Age and co-morbidities other than obesity, were not associated with long-term arthralgia. Sleep and concentration disorders were correlated with persisting joint pains, but our study design did not enable us to distinguish cause from consequence. In addition, prolonged detection of CHIKV-RNA in serum at 8 to 10 DPSO was identified as a predictor for pCHIK-CPA at 6 weeks and at 1 year.

In our study, the number of female patients was twice that of men in both confirmed CHIKV and non-CHIKV cases. We therefore postulate that the female-to-male ratio in our study reflects gender differences in health seeking behaviour, rather than genuine differences in exposure to CHIKV or frequency of symptomatic infection. The seroprevalence rates from an outbreak that involved the same CHIKV genotype Saint Martin in 2014, also showed similar rates of infection between men and women [27]. Differential susceptibility to CHIKV infection has been reported for either sex in studies from different outbreaks [70]. Female gender has been mentioned as a risk factor for long-term joint pains in many CHIKV outbreaks [48,55,71–73]. However, it has not been identified consistently, and pathophysiological evidence for a gender difference is lacking [64,74,75].

Age above 30 to 50 years was a risk factor in many reports [48,56,63,71,74–76]. In line with studies by Larrieu *et al*. and Win *et al*., that involved ECSA genotype, age did not emerge as a risk factor in our univariate analysis, which excluded participants younger than 18 years [64,73].

Obesity, a major public health problem in Aruba, predicted pCHIK-CPA. For joint pains longer than 6 weeks duration, this self-reported finding was an independent predictor of commonly associated co-morbidities such as diabetes or degenerative joint disease, which were identified as risk factors in other studies [56,63]. Obesity was also identified as an independent risk factor for CHIKV-induced arthritis in a large study from India [77]. The authors suggested that obesity may lead to a chronic inflammatory state, that is aggravated by CHIKV infection. This hypothesis merits further study, especially since others found a trend of persisting arthralgia in patients with increased body mass index, even though it did not reach statistical significance [64,75].

In contrast with previous findings, long-term arthralgia was not associated with pre-existing rheumatological disease or other comorbidities [55,56,63]. The relatively low prevalence of comorbid conditions in our cohort precludes generalization of these results.

Signs of inflammatory arthropathy and involvement of the fingers predicted long-term arthralgia in univariate, but not multivariate analysis. Instead, a high ACR-score in the acute phase was a significant risk factor for pCHIK-CPA. We therefore concur with the French guidelines for the management of chikungunya, to make an accurate assessment of the number of joints involved in the initial evaluation of CHIKV-infected patients [78].

Presence of CHIKV-RNA in serum after the first week was a strong predictor of pCHIK-CPA. This may point towards high viral loads at the time of symptom onset, which have been associated with prolonged joint pains in ECSA genotype infection [34,79]. However, another study failed to demonstrate an association between peak viral load or duration of viremia with persistent arthralgia at 6 weeks [73]. Following infection in humans, CHIKV rapidly mutates during the acute phase. Thiberville *et al*. observed that the increasing intra-host genetic diversity, with resulting higher viral amino-acid complexity in the acute phase, correlated with increased frequency of pCHIK-CPA at 300 DPSO [40]. The formation of intra-host quasi-species may help CHIKV to evade a mounting adaptive immune response, and could explain prolonged persistence of CHIKV-RNA in spite of detectable CHIKV-specific antibodies. The advent of affordable Next-Generation Sequencing instruments should enable us to investigate this hypothesis in the near future.

Finally, pCHIK-CPA was associated with extended periods of sick leave. Although mortality and hospitalization rates appear lower than those reported for the ECSA genotype outbreaks in Asia, the high attack rates of CHIKV in the Americas are associated with considerable morbidity. The increase in consultations and absenteeism we reported here, seem a crude measure of the direct and indirect health costs associated with CHIKV outbreaks.

### Limitations

Our retrospective study was subject to a number of limitations. Recall bias could not be excluded. The time lapse between the survey and disease onset might have affected acute phase reporting in particular. The structured questionnaire used multiple choice questions, which restricted the amount of detail we recorded. Further, description of inflammatory characteristics and arthralgia classification was based on self-reporting, which was reported to overestimate the number of joints involved and the presence of signs compared to assessment by a qualified physician [62]. A prospective study has been planned to assess structural joint damage in patients with persisting pCHIK-CPA in Aruba. Public awareness of lack of specific antiviral treatment probably prevented many symptomatic patients from seeking medical consultation, and may have introduced selection bias in our cohort. As with other outbreak reports, the unique interaction between infecting CHIKV strain and host population limits generalizability of our study results.

### Conclusions

We report on the first outbreak of an Asian lineage of CHIKV in Aruba. More than one fifth of cases would not have been detected if RT-PCR testing was omitted. We emphasize the need for a combined molecular and serological approach to diagnose CHIKV in a single sample during the acute phase. Rapid diagnostic tests, that meet the ASSURED criteria (affordable, sensitive, specific, user-friendly, rapid and robust, equipment-free and delivered on location) are urgently needed [80]. CHIKV-antigen detection tests are being developed, but unsatisfactory diagnostic performance across different genotypes does not permit commercialization yet [81,82].

Our survey of the clinical presentation of CHIKV infection in an adult population indicated that arthralgia, fever and skin rash were the dominant acute phase symptoms. Arthralgia was characterised by signs of inflammation in up to 71% of those affected. Forty-four percent of cases had pCHIK-CPA for longer than 6 weeks, and 26% for at least one year. In Aruba, a study has been planned to classify rheumatic patterns and to assess structural joint damage in patients with persisting pCHIK-CPA.

We identified female gender, obesity, a high ACR-score and prolonged detection of CHIKV-RNA in serum (longer than 1 week after symptom onset) as predictors for pCHIK-CPA. These risk factors should be validated in prospective studies, so they could contribute to the timely identification of CHIKV-infected patients who are likely to benefit from future therapeutic interventions, such as disease-modifying anti-rheumatic drugs or antiviral compounds [78,83–85].

## Supporting information

S1 Questionnaire The structured questionnaire form, used for collection of complementary clinical data and documentation of informed consent in a follow-up telephonic survey conducted by a qualified physician, 18–24 months after the CHIKV outbreak. (DOCX)

S1 Checklist STROBE checklist. (DOC)
