# Supplementary material for: Chikungunya virus infection in Aruba: Diagnosis, clinical features and predictors of post-chikungunya chronic polyarthralgia
Source: PLoS One. 2018 Apr 30;13(4):e0196630. doi: 10.1371/journal.pone.0196630 (PMC5927412; doi:10.1371/journal.pone.0196630)
Supplement: S1 Questionnaire — (DOCX) [file pone.0196630.s001.docx]

CHIKV ARUBA 2014-2015

**Telephone call**

*Explain why you are calling: research into 2014-15 Chikungunya epidemic with the purpose of improving diagnostics, and to find out if patients have persisting complaints.*

*Explain how the patient was found (via lab request)*

*If permission is not obtained: thank the patient for time and end the call. Record* **declined**

*Ask permission to ask questions about the above; if obtained, record* **consent obtained**

*Fill out the form below. Write clearly and encircle answers*

**Participant**

1. What is the study number? * *8 digits XLS file- to be completed by interviewer*

*_ _ _ _ _ _ _ _ DOUBLE CHECK !!*

1. What is your sex?* malefemale
2. What is your year of birth?* _ _ _ _ *The year only (4 digits)*
3. In which district do you live?*

NoordParaderaOranjestadSanta Cruz

SavanetaSan Nicolasother (outside Aruba)

1. Do you recall being tested for Chikungunya when you were ill in 2014-2015?*

YesNo

1. Did you suffer from any of the following chronic conditions before your illness?*

diabetes obesity cardiac respiratory rheumatological

osteoporosis arthrosis other none

**Questions about the acute illness when you were tested for CHIKV**

1. Did you have a fever?* Yes No
2. Did you have a rash?* Yes No
3. Did you have a headache?* YesNo
4. Did you have respiratory symptoms? Yes No **throatache, cough, dyspnea?*
5. Did you have gastro-intestinal symptoms? YesNo **Diarrhoea, vomiting, abdom. pains?*
6. Did you have neurological symptoms? YesNo **dizziness, confusion, convulsion?*

13. Did you have haemorrhagic signs? YesNo **Bruising, bleeding?*

1. Do you have sleeping problems that started after you illness?* Yes No
2. Do you have concentration problems that started after you illness?* YesNo
3. Did you have muscle pains during your illness?* YesNo
4. Did you have joint pains during your illness?* YesNo
5. Did you have other complaints related to your illness in 2014-15? *(key words only)*
6. Do you still have joint pains?* Yes No
7. Did your joint pains last 6 weeks or longer?* YesNo
8. How long did the joint pains last?*selecteer

0-2 weeks2-6 weeks6 weeks- 3 months3-6 mo 6-12 mo > 1 year

1. Which joints are/ were affected?*

shoulderelbowhipkneeanklefingerswristsfeettoesspine

1. What number of joints was affected?*

1 large joint

2-10 large joints

1-3 small joints (with or without involvement of large joints)

4-10 small joints (with or without involvement of large joints)

> 10 joints (involvement of at least 1 small joint)

*Definitions:*

*“SMALL JOINT”:MCP, PIP, MTP 2-5, thumb IP, wrist (EXCL: DIP, 1st CMC, 1st MTP)*

*“LARGE JOINT”: Shoulder, elbow, hip, knee, ankles*


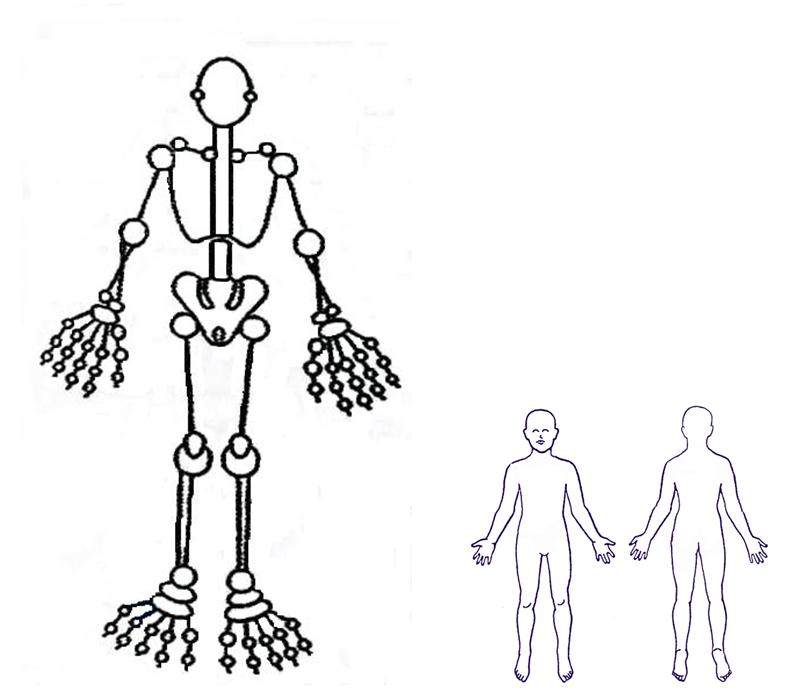


24. Has the number of painful joints changed compared to the first 6 weeks of illness?

**Do more or less joints hurt now than before?*

IncreasedDecreasedSameNot applicableI don't know

25. Did/ do you have joint redness?* YesNo

26. Did/ do you have joint swelling?* YesNo

27. Did/ do you have morning stiffness?* YesNo

**Questions about management of your illness**

**Top of Form**

**Doctor's visit, examinations, treatment**

28. Did you see your doctor after the acute illness because of persisting joint pains?*

YesNo

29. Was blood examination repeated?* YesNo

30. Were radiographs of the joints taken?* YesNo

31. Did you take any of the following drugs in the course of your illness?*select

paracetamol

NSAID_COX2

corticosteroids

DMARDS

biologicals

antibiotics

other

32. which is your botica?

**Absence from work**

33. Were you absent from work because of illness?* YesNo

34. How long have you been absent from work because of the illness?*select one answer

< 1 week1-2 weeks2-4 weeks> 4 weeksstill not able to work

Bottom of Form
